# Persistent Intracellular *Staphylococcus aureus* in Keratinocytes Lead to Activation of the Complement System with Subsequent Reduction in the Intracellular Bacterial Load

**DOI:** 10.3389/fimmu.2018.00396

**Published:** 2018-03-01

**Authors:** Anas H. Abu-Humaidan, Malin Elvén, Andreas Sonesson, Peter Garred, Ole E. Sørensen

**Affiliations:** ^1^Department of Clinical Sciences Lund, Infection Medicine, Lund University, Lund, Sweden; ^2^Skåne University Hospital, Department of Clinical Sciences Lund, Dermatology and Venereology, Lund University, Lund, Sweden; ^3^Laboratory of Molecular Medicine, Department of Clinical Immunology Section 7631, Copenhagen University Hospital, Rigshospitalet, Copenhagen, Denmark; ^4^Leo Pharma A/S, Ballerup, Denmark

**Keywords:** complement activation, membrane attack complex, classical pathway activation, intracellular infection, *Staphylococcus aureus*, atopic dermatitis, Erk activation

## Abstract

The complement system is an ancient part of the innate immune system important for both tissue homeostasis and host defense. However, bacteria like *Staphylococcus aureus* (SA) possess elaborative mechanisms for evading both the complement system and other parts of the immune system. One of these evasive mechanisms—important in causing chronic and therapy resistant infections—is the intracellular persistence in non-immune cells. The objective of our study was to investigate whether persistent intracellular SA infection of epidermal keratinocytes resulted in complement activation. Using fluorescence microscopy, we found that persistent SA, surviving intracellularly in keratinocytes, caused activation of the complement system with formation of the terminal complement complex (TCC) at the cell surface. Skin samples from atopic dermatitis patients analyzed by bacterial culture and microscopy, demonstrated that SA colonization was associated with the presence of intracellular bacteria and deposition of the TCC in epidermis *in vivo*. Complement activation on keratinocytes with persistent intracellular bacteria was found with sera deficient/depleted of the complement components C1q, Mannan-binding lectin, or complement factor B, demonstrating involvement of more than one complement activation pathway. Viable bacterial counts showed that complement activation at the cell surface initiated cellular responses that significantly reduced the intracellular bacterial burden. The use of an inhibitor of the extracellular signal-regulated kinase (ERK) abrogated the complement-induced reduction in intracellular bacterial load. These data bridge the roles of the complement system in tissue homeostasis and innate immunity and illustrate a novel mechanism by which the complement system combats persistent intracellular bacteria in epithelial cells.

## Introduction

Encompassing more than 30 proteins, the complement system is an ancient part of innate immunity (1), and it was originally described for its ability to complement phagocytes and antibodies in microbial killing (2). The complement system combats infections by detection of microbial intruders followed by lysis or opsonization to facilitate microbial clearance. In tissue homeostasis, the complement system plays a role as an intricate surveillance system by detecting altered host cells and facilitates clearance of dead or altered cells (1, 3). The different roles of the complement system are illustrated by complement deficiencies, which increase susceptibility to bacterial infections and/or autoimmune diseases, for example, C3 deficiency is generally associated with increased risk of bacterial infections, while deficiency of late complement component C5–C9 is associated with Neisseria infections in particular (4). Deficiency of C1q, which is important for clearance of apoptotic cells, is associated with systemic lupus erythematosus (5).

*Staphylococcus aureus* (SA) is an important human pathogen and a major cause of soft tissue, respiratory, bone, joint, and endovascular disorders (6, 7). SA is commonly associated with skin infections (8), for example in diseases like atopic dermatitis (AD) (9), a chronic inflammatory pruritic skin disease characterized by dry skin, pruritus, erythema, edema, scaling, excoriations, oozing, and lichenification (9). Disease flares of AD are associated with a microbial dysbiosis with abundance of SA (10, 11) suggested to drive inflammation (12).

*Staphylococcus aureus* possess elaborative mechanisms for shielding against complement mediated killing (13) among its impressive arsenal of immune evasive strategies (14). One important immune evasive mechanism by which SA is suggested to cause chronic and therapy-refractive infections is by intracellular survival in viable immune and non-immune host cells (15–21). SA can survive intracellularly in non-immune cells by switching phenotype to small colony variants (SCVs), a phenotype that does not invoke the same host responses in the cells as the wild type (9, 22, 23). This phenotype has been implicated in failure of antibiotic treatments (24). In AD, the dynamic interplay between SA and keratinocytes is proposed to result in a selection for bacteria that stimulate autophagy, thereby promoting degradation of the inflammasome and consequently promoting intracellular bacterial survival (25).

Immune responses to extracellular SA infections are extensively studied, while the immune responses to persistent intracellular bacteria in non-professional immune cells like keratinocytes, despite its clinical importance, are poorly understood. Since persistent intracellular bacteria presumably result in changes of cellular homeostasis, we investigated whether persistent intracellular SA in epidermal keratinocytes could alert the body’s intricate surveillance system, the complement system.

In the current study, we found that persistent intracellular bacteria surviving in epidermal keratinocytes promoted complement activation on the cell surface, this complement activation in turn initiated cellular responses that subsequently reduced the intracellular bacterial burden by an extracellular signal regulated kinase (ERK) dependent mechanism. To the best of our knowledge, this demonstrates for the first time a possible role of the complement system in combating persistent intracellular bacteria in non-phagocytic epithelial cells, thus, bridging the dual roles of the complement system in tissue homeostasis and host defense.

## Materials and Methods

### Reagents

Rabbit polyclonal antibody against the C3d domain of human complement component 3 (C3), and rabbit polyclonal antibody against the C4c domain of human complement component 4 (C4) were purchased from Dako. Mouse monoclonal anti human C5b-9 antibody directed against a neoepitope exposed on complement component 9 when incorporated into the terminal complement complex (TCC) is from BioPorto Diagnostics. Mouse monoclonal against human complement component 1q (C1q) is from Quidel. Rabbit polyclonal anti SA antibody is from Thermoscientific. Alexa fluor 488 goat anti rabbit IgG, Alexa fluor 594 goat anti mouse IgG and Alexa fluor 488 goat anti human from Molecular Probes. U0126, CRID-3, and AG1478 all used at concentration of (10 µM) from Tocris. Human purified C1q, Mannan-binding lectin (MBL), complement Factor B, C3, C6 proteins and sera depleted of C1q, C3, Factor B, C6 were from Quidel. The MBL deficient serum was obtained from an individual homozygous for the D (R52C, rs5030737) variant not able to activate the MBL dependent lectin pathway has been previously described (26). IdeS was purified as previously described (27) and was generously provided by Lars Björck and Inga-Maria Frick.

### Cell Culture and Inhibition Assays

Keratinocytes were cultured as previously described (28) to near confluence in KGM medium (Lonza) with additional EGF (100 ng/ml). A day before confluence the medium was changed to KGM without EGF or insulin for 24 h to induce differentiation, and then changed to KGM without EGF, insulin or antibiotics for another 24 h before starting the infection experiments. For some experiments, confluent cells were treated for 48 h with the epidermal growth factor receptor (EGFR) inhibitor AG1478 (10 µM), and the medium was changed to KGM without EGF, insulin, or antibiotics overnight before starting the infection assays. For inhibition of ERK pathway signaling, medium containing 10 µM U0126 was used during complement activation on infected cells and until processing of cells for viable count. For inflammasome inhibition, medium containing 10 µM CP-456,773 (CRID-3) was used during complement activation and until processing of infected cells for viable counts. Both inhibitors were solubilized in DMSO and used at the same volume, and viability of cells following treatment of both inhibitors was checked using microscopy and trypan blue staining (described below).

### Human Skin Samples

Atopic dermatitis patients aged 18 years or over, with AD verified by the UK refinement of the Hanifin and Rajka diagnostic criteria for AD (29, 30), were recruited from the Dermatology Clinic at Lund University Hospital, Lund, Sweden. Tissue biopsies from AD lesional areas were taken from the skin of the patients. The participants gave informed consent complying with the Helsinki Declaration, and the Regional Ethics Examination Board of Lund, Sweden approved the study (Permit Numbers: 144/2010, 317/2010, 82/2012).

### Immunohistochemistry of Skin Samples

The skin specimens were fixed in 4% formaldehyde, dehydrated, and embedded in paraffin. Slices (4 µm) were made and placed on superfrost plus slides (Thermofisher), followed by incubation at 60°C for 1 h. The slides were then processed in PT link module (Dako) for deparaffinization, dehydration, and epitope retrieval. EnVision FLEX Target Retrieval Solution, High pH (Dako) was used for this process at 97°C for 20 min. After Ag retrieval, slides were blocked for 1 h at room temperature using a blocking solution of TBS with 0.05% Tween 20 (TTBS), 1% BSA, and 5% serum from the same species as the secondary Abs were raised. The slides were then incubated overnight with primary Abs diluted 1:500 in the same blocking solution for 24 h. The slides were washed three times for 20 min in TTBS and incubated for 24 h with secondary Abs diluted 1:1,000 in the same blocking solution. The slides were then washed again three times and mounted with Prolong Gold antifade reagent mounting medium with DAPI (Invitrogen).

### Bacterial Culture and Intracellular Infection Assay

The bacterial culture and intracellular infection experiments were performed essentially as previously described (31). An invasive clinical isolate of SA from atopic eczema (2957/13) and SA Newman were plated on Todd Hewitt with yeast (THY) agar and subsequently cultured overnight in THY broth, washed and used for experiments. Confluent keratinocytes in antibiotic free medium were infected with a multiplicity of infection (MOI) of 10–20 in 12 and 24-well plates, the plates were centrifuged at 1,000×*g* for 2 min to enhance uniformity of SA attachment to keratinocytes and incubated at 37°C for 3 h. Medium was then aspirated and changed to medium containing 100 µg/ml gentamicin for 90 min to kill extracellular bacteria. Medium containing 10 µg/ml gentamicin was used throughout the remaining of the experiment.

For “*intracellular SA present immediately after infection*” keratinocytes were lysed for bacterial viable count after 90 min with 100 µg/ml gentamicin. For “persistent intracellular SA” keratinocytes were cultured for an additional 24 h in medium with 10 µg/ml gentamicin.

### Complement Activation Assay

Complement activation on keratinocytes was performed as described (28). Briefly, after 0 or 24 h in medium containing 10 µg/ml gentamicin, infected, or control keratinocytes were incubated with 10% normal human serum (NHS), heat inactivated human serum (HIS), depleted serum or medium for 3 h, as a source of complement. Cells were consequently washed in PBS and processing of keratinocytes for intracellular bacterial viable counts or fluorescence microscopy was done either directly or 24 h after addition of the different sera.

### Intracellular Bacterial Viable Counts

Keratinocytes were washed three times in ice cold PBS, scrapped, and lysed in 0.1% Triton X-100 in sterile water and vortexed several times. Lysates were serially diluted and plated on THY agar plates and colony forming units counts were performed the next day as described (31).

### Immunofluorescence (IF) Microscopy

Keratinocytes grown on inserts in 12 well plates were washed three times in ice cold PBS and fixed for 45 min in 4% PFA at room temperature. After two washes in PBS, the cells were blocked with 5% goat serum and 5 mg/ml BSA at room temperature for 45 min in PBS with 0.05% Tween 20 (PBST). After blocking, incubation was performed with primary antibodies diluted in PBST with 2.5% goat serum and 5 mg/ml BSA overnight in cold room under rotation. Next day, inserts were washed three times in PBST and incubated with secondary antibodies for 2–4 h at room temperature. The inserts were washed three times and mounted on slides using Prolong Gold antifade reagent mounting medium with DAPI (Invitrogen). Samples were visualized using a Nikon Ti-E microscope (Nikon) inverted fluorescence microscope equipped with Andor Neo/Zyla camera (Andor) and NIS elements advanced research software (Nikon) and a Plan Apochromat objective (Olympus). Fluorescence quantification was done by acquiring several images of each monolayer, covering around 10,000 cells, then analyzed with IntDen measurement (the product of Area and Mean Gray Value) using Fiji (32). No primary antibody controls are used as a reference for quantification.

### Detection of Apoptosis and Necrosis

Apoptotic and necrotic cells were detected using an annexin V-FITC/Ethidium homodimer III (EtD-III) staining kit (Biotium), according to the manufacturer’s protocol. Briefly, control and infected cells were washed twice with PBS then incubated with appropriate dilutions of annexin V-FITC and EtD-III in binding buffer for 30 min. Washed twice in binding buffer and fixed with 4% PFA containing 1.25 mM calcium chloride (CaCl_2_) at room temperature for 30 min, washed three times in PBS containing 1.25 mM CaCl_2_ and mounted on slides using Prolong Gold antifade reagent mounting medium with DAPI. Viability of infected cells treated with U0126 and CRID-3 was assessed using Trypan blue staining, briefly, cells were washed twice with PBS and incubated with trypsin for 10 min, cell suspension was then mixed 1:1 (v/v) with 0.4% trypan blue solution (Amresco) for 5 min, and viability was assessed using LUNA Automated Cell Counter (Logo Biosystems).

### SDS-PAGE and Immunoblotting

To examine the medium of control and infected cells for degradation of complement components, medium was collected and centrifuged at 10,000 *g* for 10 min to remove cell debris, and supernatant was precipitated using 10% trichloroacetic acid. SDS-PAGE and immunoblotting were performed on the precipitated medium according to the instructions from the manufacturer (Bio-Rad). After transfer of proteins from the polyacrylamide gels, the polyvinylidene difluoride (PVDF) membrane was fixed for 30 min in TBS with 0.05% glutaraldehyde (Sigma-Aldrich) and blocked with 3% skimmed milk in TTBS for 30 min. PVDF membranes were then incubated overnight with primary antibody diluted in blocking solution. The following day, the membranes were incubated for 2 h with HRP-conjugated secondary antibody in blocking solution (Jackson Immunoresearch) and visualized by SuperSignal West Pico Chemiluminescent Substrate (Pierce). The PVDF membrane was stripped for 20 min in 0.2 mol/l glycine (pH 2.5) and 1% SDS, washed twice with TTBS, and finally blocked before incubating overnight with a new antibody.

### Real-time PCR

RNA was purified from control and infected cells using Direct-zol RNA miniprep (zymo research) according to the manufacturer instructions. cDNA was synthesized from 200 ng purified RNA using iScript cDNA synthesis kit (Bio-Rad), according to the instructions given by the manufacturer. RNA expression of complement components was analyzed with quantitative RT-PCR using iQ SYBR Green Supermix (Bio-Rad). Amplification was performed at 55°C for 40 cycles in iCycler Thermal Cycler (Bio-Rad), and data were analyzed using iCycler iQ Optical System Software. RNA expression was normalized using GADPH as housekeeping gene.

### Statistical Analysis

Student’s *t*-test was performed on log transformed values to compare different treatments in the case of viable counts. While for fluorescence quantification, Student’s *t*-test was performed on non-log transformed values. * denotes *p* < 0.05, ** denotes *p* < 0.01.

## Results

### Persisting Intracellular SA Cause Deposition of Complement Fragments on Primary Keratinocytes

**Figure 1:**
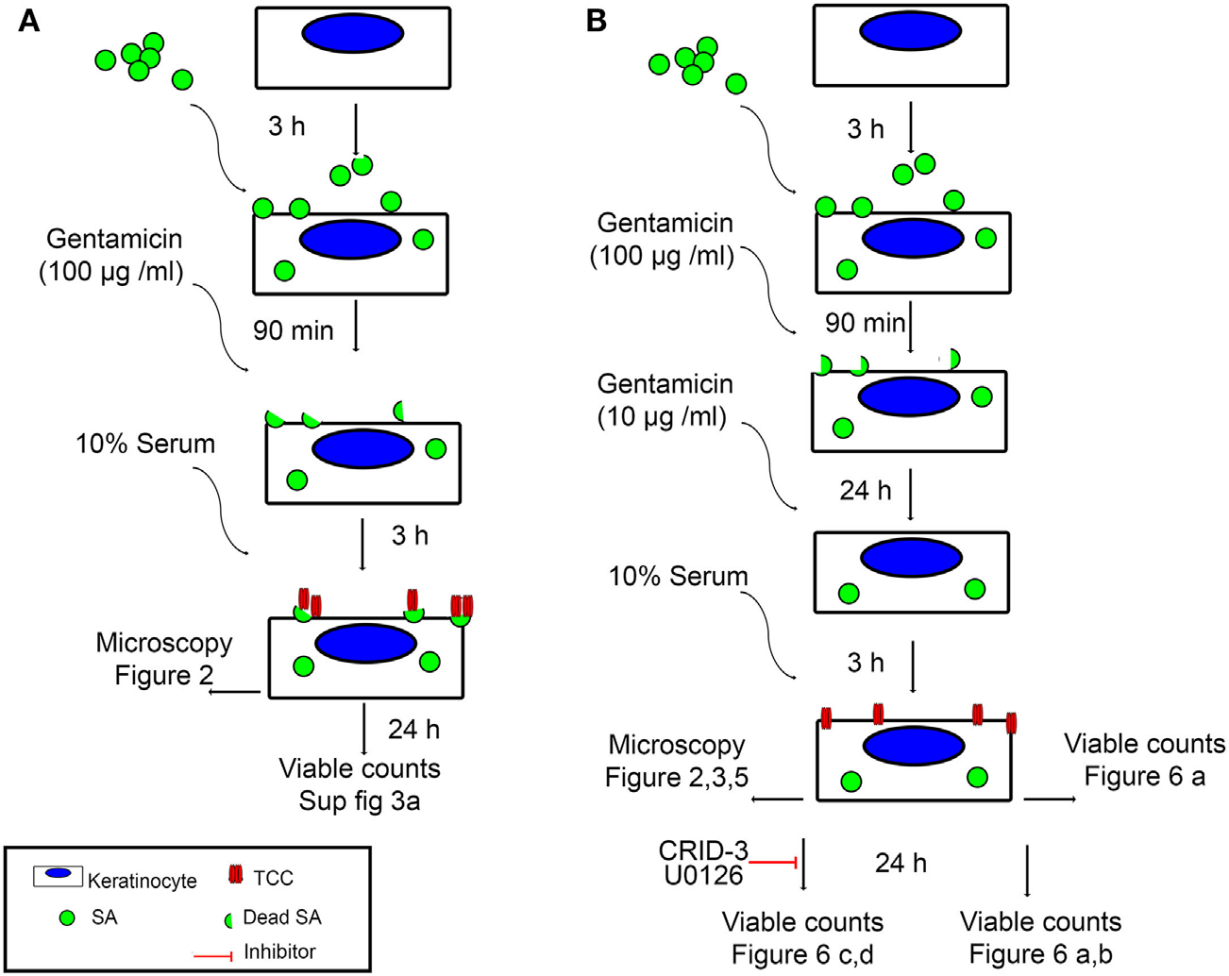
Experimental setup. An illustration of the two infection models used in this study, as well as the figures corresponding to the time points and analysis methods used. **(A)** Keratinocyte monolayers are infected with *Staphylococcus aureus* (SA) for 3 h, followed by killing of extracellular bacteria using 100 µg/ml Gentamicin for 90 min. At this time point, intracellular SA is referred to in the text as *intracellular SA present immediately after infection*. Incubation with normal human serum (NHS) at this time point leads to deposition of terminal complement complex (TCC) mostly on extracellular bacterial remnants. **(B)** Keratinocyte monolayers are infected with SA for 3 h, followed by killing of extracellular bacteria using 100 µg/ml Gentamicin for 90 min, keratinocytes are further incubated with 10 µg/ml Gentamicin for 24 h, at this time point, intracellular SA is referred to in the text as *persistent intracellular SA*. Incubation with NHS at this time point leads to deposition of TCC on the surface of keratinocytes.

To investigate if persistent intracellular survival of bacteria in non-immune cells results in complement activation at the cell surface, we set up a model of persisting intracellular infection with SA in epidermal keratinocytes. Primary epidermal keratinocytes were infected with an invasive strain of SA isolated from the skin of an AD patient. Cells were infected one day after confluence was reached, to ensure stable cell number throughout the experiment and to mimic the upper epidermal layer. After 3 h of infection, extracellular bacteria were killed using gentamicin (100 µg/ml) for 90 min, while intracellular SA remained viable (33). At this time point (T0), SA will be described as “*intracellular SA present immediately after infection*” in our model. Keratinocytes were then maintained in a medium containing gentamicin (10 µg/ml) for 24 h (T24). This time point where viable intracellular bacteria have persisted for more than 24 h we termed “*persistent intracellular SA*.” The infection model is illustrated in Figure 1.

**Figure 2:**
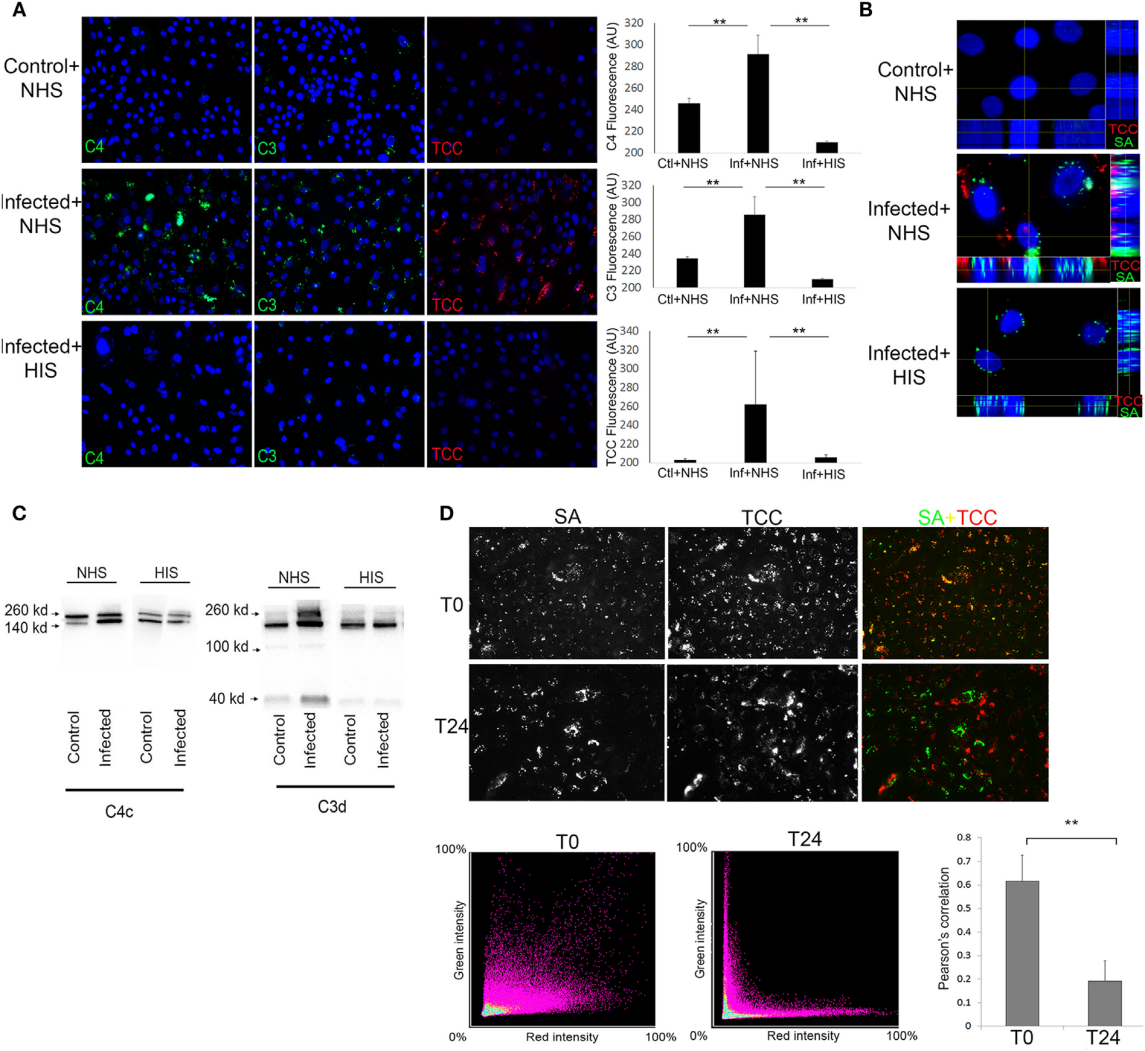
Keratinocytes with persistent intracellular *Staphylococcus aureus* (SA) activate the complement system on the cell surface. Immunofluorescence (IF) microscopy of primary keratinocytes infected with intracellular SA. **(A)** Keratinocytes were stained for complement activation fragments component 4 (C4), component 3 (C3), and terminal complement complex (TCC) using normal human serum (NHS) or HIS as a source of complement, IF was quantified in the panel to the right. **(B)** Orthogonal view of Z-stacks of keratinocytes infected with intracellular SA (green) show deposition of TCC (red) when incubated with NHS but not HIS. **(C)** New medium was added to the cells after complement activation and collected after 24 h to monitor release of activation fragments deposited on cells, Western blots show higher signal of C3 and C4 degradation products in media of infected cells treated with NHS. **(D)** Keratinocytes with intracellular SA present immediately after infection (T0) or persistent intracellular SA (T24) were incubated with NHS, colocalization (yellow) of SA (green) and TCC (red) indicates complement deposition on extracellular SA, colocalization was quantified over three experiments and represented using Pearson’s correlation coefficient in the bar graph in the lower right corner.

At T24 keratinocytes with *persistent intracellular SA* were incubated with 10% Normal human serum (NHS) as a source of complement. Using fluorescence microscopy, we found deposition of the TCC on keratinocytes with *persistent intracellular SA* (Figures 2A,B), this was accompanied by increased deposition of complement C3 and complement C4 on keratinocytes with *persistent intracellular SA* in comparison to non-infected keratinocytes (Figure 2A). Keratinocytes with *persistent intracellular SA* incubated with heat-inactivated serum (HIS) lacking complement activity did not show deposition of C3, C4, or TCC (Figures 2A,B). Western blots of conditioned medium collected after 24 h of NHS treatment of keratinocytes with *persistent intracellular SA*, showed increased C3 and C4 degradation in medium compared to controls (Figure 2C). The combined data of C3, C4, and TCC deposition, along with increased C3 and C4 degradation products in the medium, confirmed that keratinocytes with *persistent intracellular SA* activated the complement system.

To rule out participation of surviving extracellular SA, medium was plated both at T0 and T24, where no viable SA were found. To demonstrate that complement activation was not due to extracellular bacterial remnants, NHS was added either to keratinocytes with *intracellular SA present immediately after infection* (T0) or to keratinocytes with *persistent intracellular SA* (T24) to compare the pattern of complement deposition. In keratinocytes with *intracellular SA present immediately after infection* at T0, colocalization of TCC and SA was found by IF (Figure 2D) demonstrating that complement activation was associated with bacterial remnants present extracellularly. In contrast, only limited colocalization of complement and SA was found when NHS was added to keratinocytes with *persistent intracellular SA* at T24. This indicated that complement activation on keratinocytes with *persistent intracellular SA* was not associated with extracellular bacterial remnants (Figure 2D). This was further substantiated using *SA Newman*, a strain defective in host cell invasion (34). We found colocalization of SA and TCC staining even at T24 (Figure S1 in Supplementary Material), indicating that colocalization of staining determines deposition of complement on extracellular bacterial remnants. Taken together, these data demonstrate that complement activation elicited by keratinocytes with *persistent intracellular SA* was not due to the mere presence of extracellular bacteria or bacterial remnants, but possibly involved cellular changes/responses due to the presence of the persistent intracellular bacteria.

### Activation of the Complement System by Keratinocytes with *Persistent Intracellular Bacteria* Involves More than One Activation Pathway

**Figure 3:**
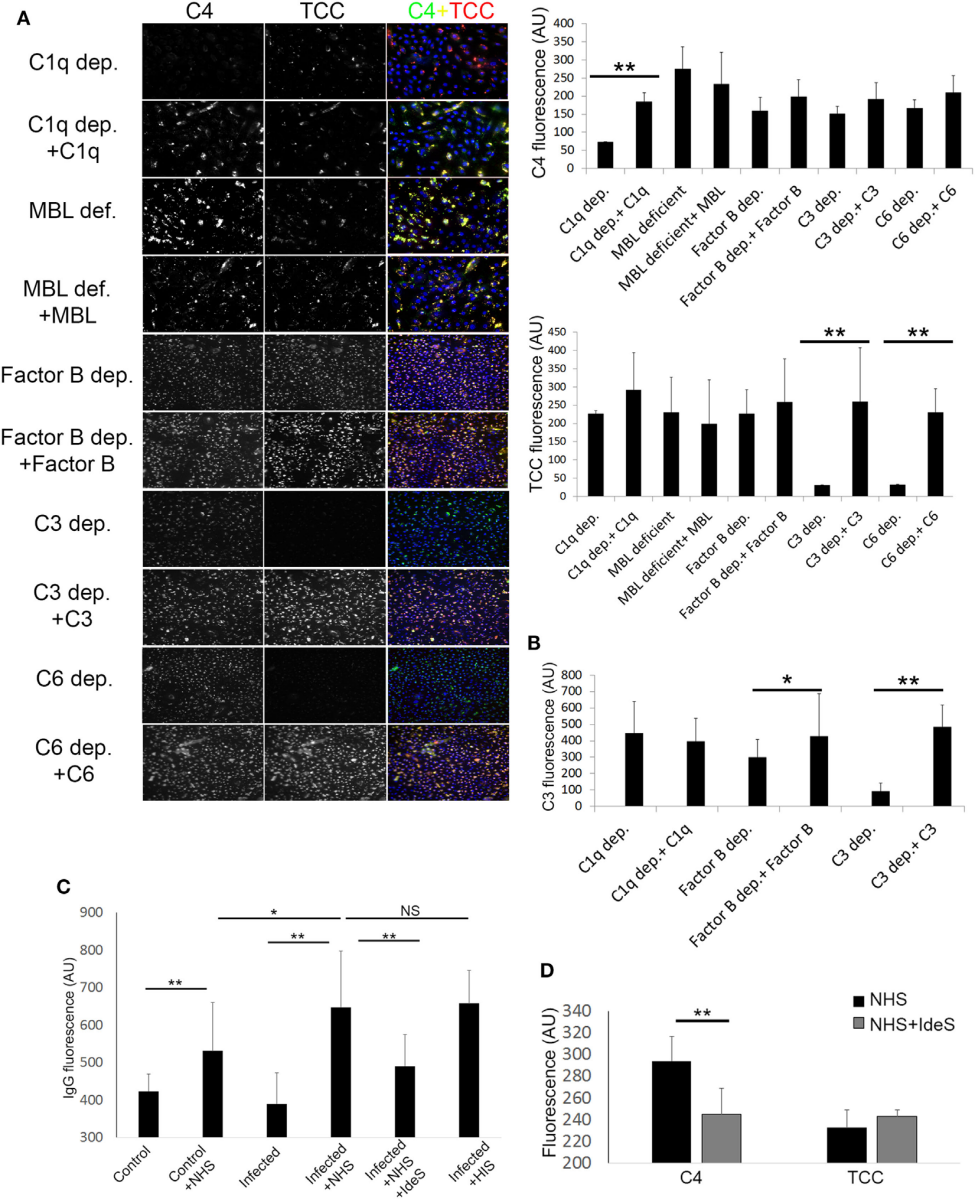
Pathways involved in complement activation induced by Keratinocytes with persistent intracellular *Staphylococcus aureus*. **(A)** Infected keratinocytes were incubated with depleted or deficient sera along with respective reconstitution proteins as a source of complement, then stained for component 4 (C4) and terminal complement complex (TCC). Immunofluorescence (IF) representative images are shown in the left panel along with quantification on the right. **(B)** IF of C3 was quantified in different depleted and reconstituted sera to confirm participation of the alternative pathway. **(C)** IF of IgG was quantified using anti human IgG antibodies, binding of IgG was found to be increased in infected keratinocytes incubated with normal human serum (NHS) or HIS. Treatment of NHS with IdeS significantly decreased IgG binding. **(D)** IF C4 and TCC were quantified in infected keratinocytes incubated with NHS or IdeS treated NHS.

By using sera depleted or deficient of essential complement components, along with C3, C4, and TCC IF staining, we investigated the pathways involved in complement activation initiated by keratinocytes with *persistent intracellular SA*. We found that complement C1q depleted, and MBL deficient sera, both activated the terminal pathway as shown by TCC staining (Figure 3A), and reconstitution of both C1q depleted and MBL deficient sera did not cause significant increase in TCC staining (Figure 3A). However, reconstitution of C1q depleted serum with C1q significantly increased C4 staining, unlike reconstitution of MBL deficient serum with MBL (Figure 3A). This indicated that the classical pathway is a major contributor to the C4 deposition. To investigate the contribution of the alternative pathway, we used Factor B depleted serum. Factor B depleted serum activated the terminal pathway, and reconstitution with complement factor B did not significantly alter C4 or TCC staining (Figure 3A). However, unlike reconstitution of C1q depleted serum, reconstitution of Factor B depleted serum increased C3 deposition (Figure 3B), indicating a role for the alternative pathway in propagation of C3 activation. As expected, C3 and C6 depleted sera did not give rise to TCC deposition on keratinocytes with *persistent intracellular SA* unless reconstituted (Figure 3A), and reconstitution of C3 depleted serum increased C3 deposition (Figure 3B).

Since immune complexes are known to activate complement through the classical pathway (35), we assessed binding of natural IgG to keratinocytes with *persistent intracellular SA* by IF. We found increased IgG staining in keratinocytes with *persistent intracellular SA* compared to non-infected keratinocytes when incubated with NHS. The IgG staining was significantly reduced if NHS was treated with IdeS, a specific streptococcal IgG degrading cysteine proteinase (27)—before incubation with keratinocytes (Figure 3C). IdeS treated NHS caused a decrease in C4 staining compared to non-treated NHS, but terminal pathway activation was not significantly reduced as shown by TCC staining (Figure 3D).

In aggregate, these data suggest a redundancy in activation of the complement system by keratinocytes with *persistent intracellular SA*, since C1q depleted, MBL deficient and Factor B depleted sera all gave rise to deposition of TCC. The classical pathway played a major role in C4 deposition as absence of C1q or degradation of IgG significantly decreased C4 staining, while the alternative pathway seemed to play a role in the propagation of C3 activation.

### Complement Is Activated in Epidermis Colonized with SA *In Vivo*

**Figure 4:**
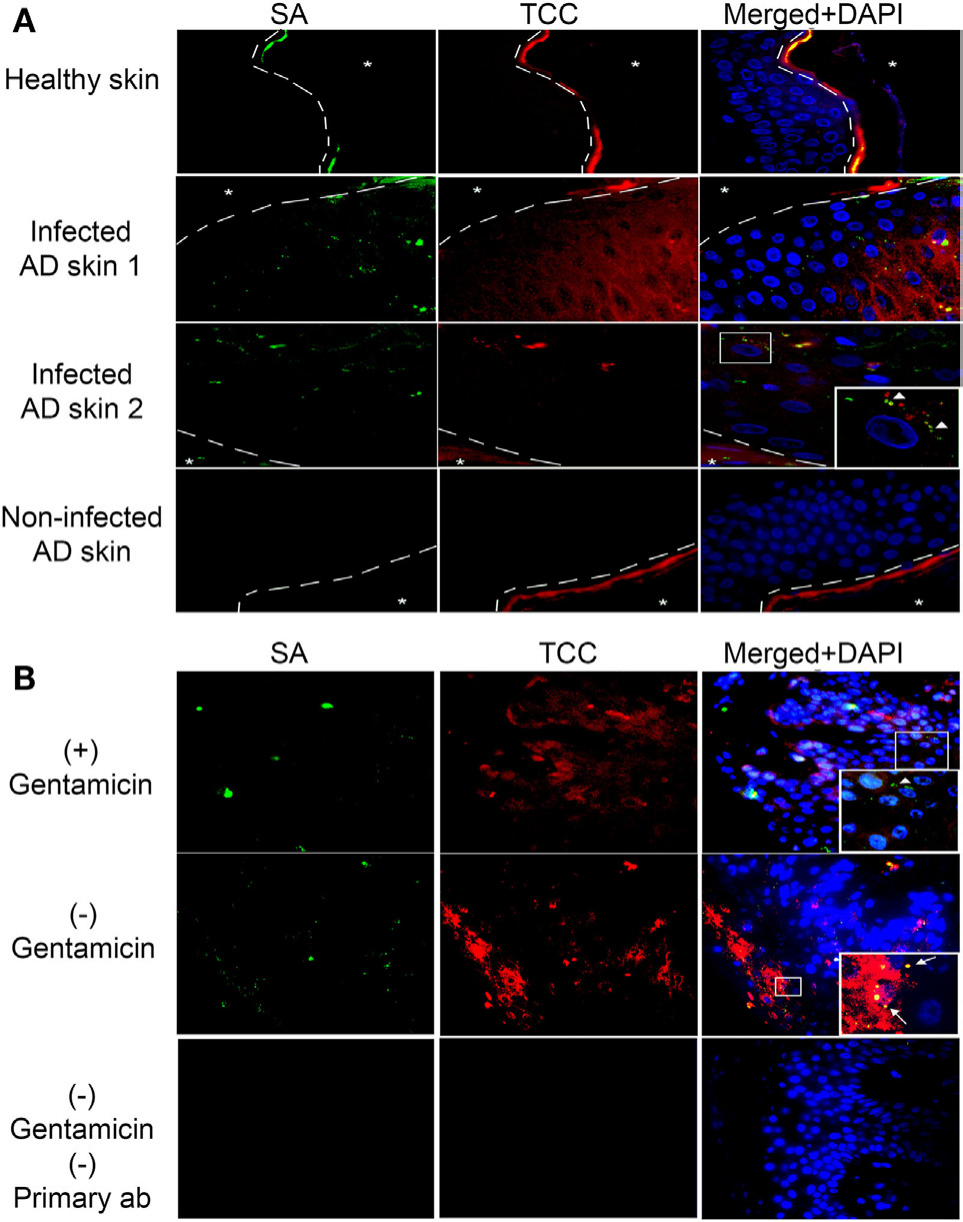
Complement activation in *Staphylococcus aureus* (SA) colonized atopic dermatitis (AD) epidermis. Representative immunofluorescence images of skin samples from healthy and AD patients stained for SA and terminal complement complex (TCC). **(A)** SA colonized AD epidermis shows an increase in TCC staining near infected keratinocytes, in comparison to healthy epidermis, or AD epidermis not colonized with SA. SA and TCC staining colocalized in some instances (arrow heads in inset), indicating deposition on extracellular bacteria, while in other instances it did not, suggesting intracellular localization of SA. **(B)** To confirm intracellular presence of SA, SA-infected AD epidermis was treated with gentamicin for 24 h to kill extracellular SA or left untreated, then incubated with normal human serum (NHS). Gentamicin treated epidermis showed SA staining, suggesting presence of intracellular SA (arrow heads in inset), while TCC staining did not colocalize with SA to the degree found in non-treated epidermis, which showed increased SA and TCC colocalization (arrows in inset). A no primary control is used for comparison of staining. Asterixis represent the apical side of the epidermis, which is not included in the investigation behind the dashed line due to the high unspecific binding of keratin.

Atopic dermatitis is a chronic inflammatory skin disease and AD patients are commonly colonized with SA during AD flares (9). To investigate if complement activation takes place on epidermal keratinocytes infected with intracellular SA *in vivo*, we double-stained with IF the epidermis of five AD patients with or without SA colonization and three healthy controls for SA and TCC. We found increased staining of TCC in SA colonized AD epidermis both in comparison to healthy controls and AD epidermis not colonized with SA (Figure 4A), demonstrating that SA colonization was associated with complement activation in AD. In some instances, the staining of TCC and SA co-localized, suggesting TCC formation on extracellular SA (arrow heads in inset, Figure 4A). In other instances, we found a similar pattern as in our model with *persistent intracellular SA* in keratinocytes where TCC and SA did not colocalize, suggesting that intracellular SA in epidermal keratinocytes may contribute to complement activation (Figure 4A).

To substantiate the presence of intracellular SA in AD skin, SA-colonized AD skin (verified by growth of SA colonies on agar) was incubated overnight in medium with or without gentamicin (10 µg/ml) to kill extracellular SA, and then incubated in 10% NHS as a source of complement before processing tissue for IF. Intracellular presence of SA in AD epidermis was demonstrated by the positive staining of SA in AD skin after gentamicin treatment (Figure 4B, arrow head in inset). The SA staining in the gentamicin treated skin did not strictly co-localize with TCC staining (Figure 4B), thus, displaying a similar staining pattern as found in our model of keratinocytes *with persistent intracellular SA*. Non-gentamicin treated skin, in which extracellular and intracellular SA was present, demonstrated more generalized co-localization of SA and TCC compared to gentamicin treated skin (Figure 4B, arrows in inset), as well as more SA and TCC staining in general (Figure 4B). These data indicate that intracellular SA is present in keratinocytes of SA colonized AD epidermis and could possibly play a role in activating complement *in vivo*.

### Complement Activation Takes Place on Viable Keratinocytes

**Figure 5:**
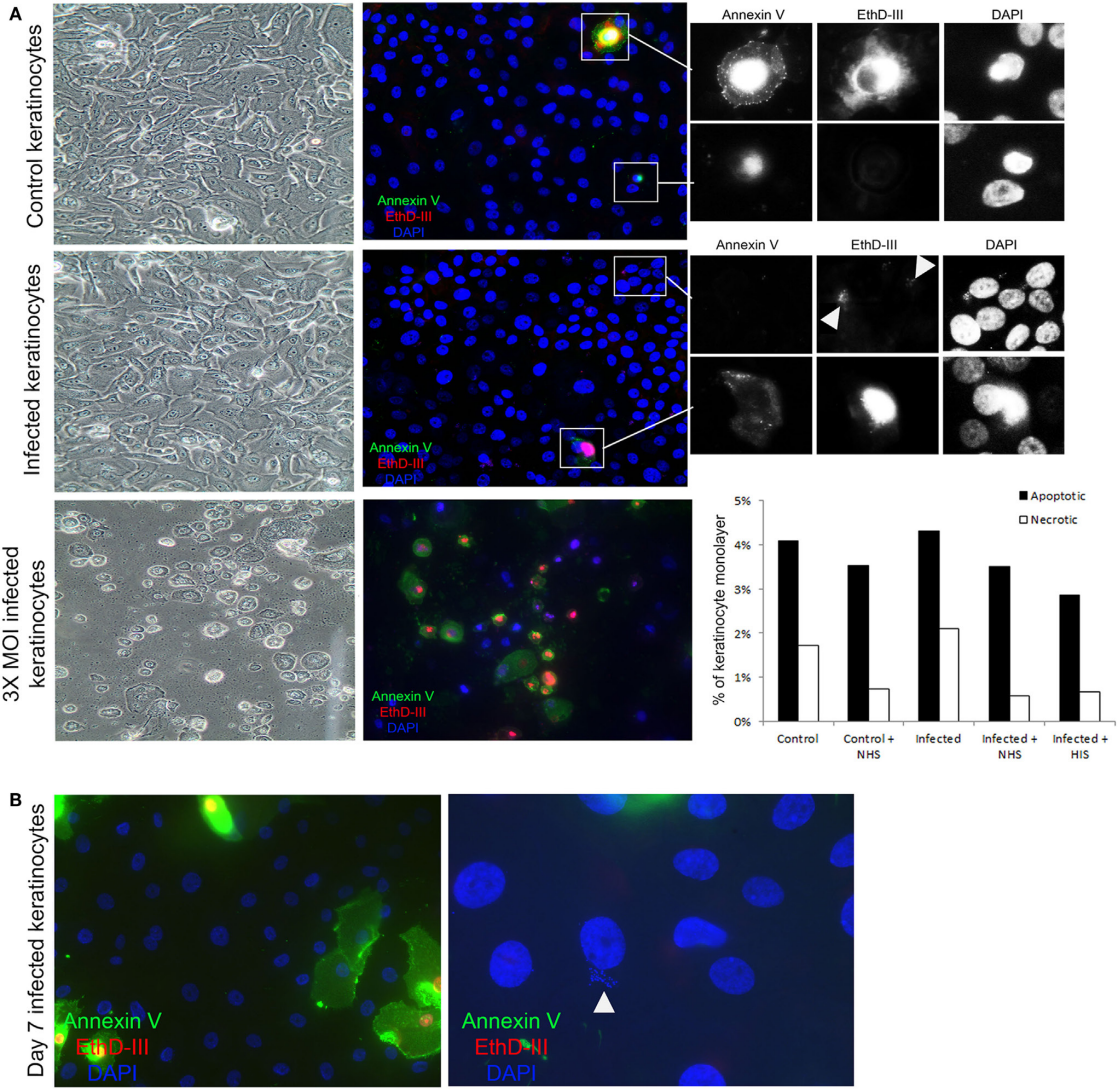
Complement activation takes place on viable keratinocytes. **(A)** Immunofluorescence microscopy of control and infected keratinocytes shows no significant difference in apoptotic cells stained with Annexin V (green), and necrotic cells with EthD-III (red). A representative experiment was quantified in the bar graph to the right, treatment with normal human serum (NHS)/HIS did not show any significant difference. Insets show a close up of apoptotic and necrotic cells. Arrow heads point to *Staphylococcus aureus* DNA stained with EthD-III. Light microscopy shows intact monolayers of control and infected primary keratinocytes. A higher multiplicity of infection (MOI) induced extensive apoptosis and necrosis in the monolayers and was used as a positive control. **(B)** Keratinocytes infected for 7 days were found to harbor intracellular bacteria, both keratinocytes and bacteria (arrow head) shown in the image are thought to be viable since they did not stain for the apoptosis or necrosis markers.

To investigate whether the observed complement activation was due to cell death, we used an apoptosis and necrosis quantification kit, that employs annexin V binding to the membrane of apoptotic cells (36), and EtD-III to selectively stain necrotic cells (37). We found no significant difference in apoptosis and necrosis between keratinocytes with or without *persistent intracellular SA* in our experimental model (Figure 5A). Moreover, morphological hallmarks of apoptosis (cell shrinkage and nuclear fragmentation) were absent in the keratinocytes with *persistent intracellular SA* (Figure 5A). This indicated that complement activation occurs in viable keratinocytes. When increasing the MOI three times compared to the MOI used in our study, there was significant apoptosis and necrosis in the monolayer (Figure 5A), in accordance with other studies showing that bacterial load affects the cellular survival upon infection (38).

### Complement Activation Is Found When Persistent Intracellular SA Were Present in SCVs

In our model of *persistent intracellular SA*, we did not observe SCVs commonly implicated in chronic and antibiotic resistant SA infections (20). However, SA survived for up to 7 days in viable keratinocytes (Figure 5B), but after 7 days it was mostly in the form of SCVs, as judged by the morphology of the colonies on agar. Increased apoptosis and necrosis (Figure S2A in Supplementary Material) was observed 7 days after the time the cells were infected, both in keratinocytes with and without *persistent intracellular SA*, probably due to the normal life cycle of the confluent culture of primary keratinocytes. At the time point where SCVs were present intracellularly, incubation with NHS still resulted in increased TCC deposition in cells with *persistent intracellular SA* in comparison to controls with no intracellular bacteria (Figure S2B in Supplementary Material). This demonstrated that *persistent intracellular SA* promoted complement activation in viable keratinocytes even when the intracellular bacteria were present as SCVs after prolonged intracellular survival.

### Complement Activation Leads to a Reduction of Persistent Intracellular SA

**Figure 6:**
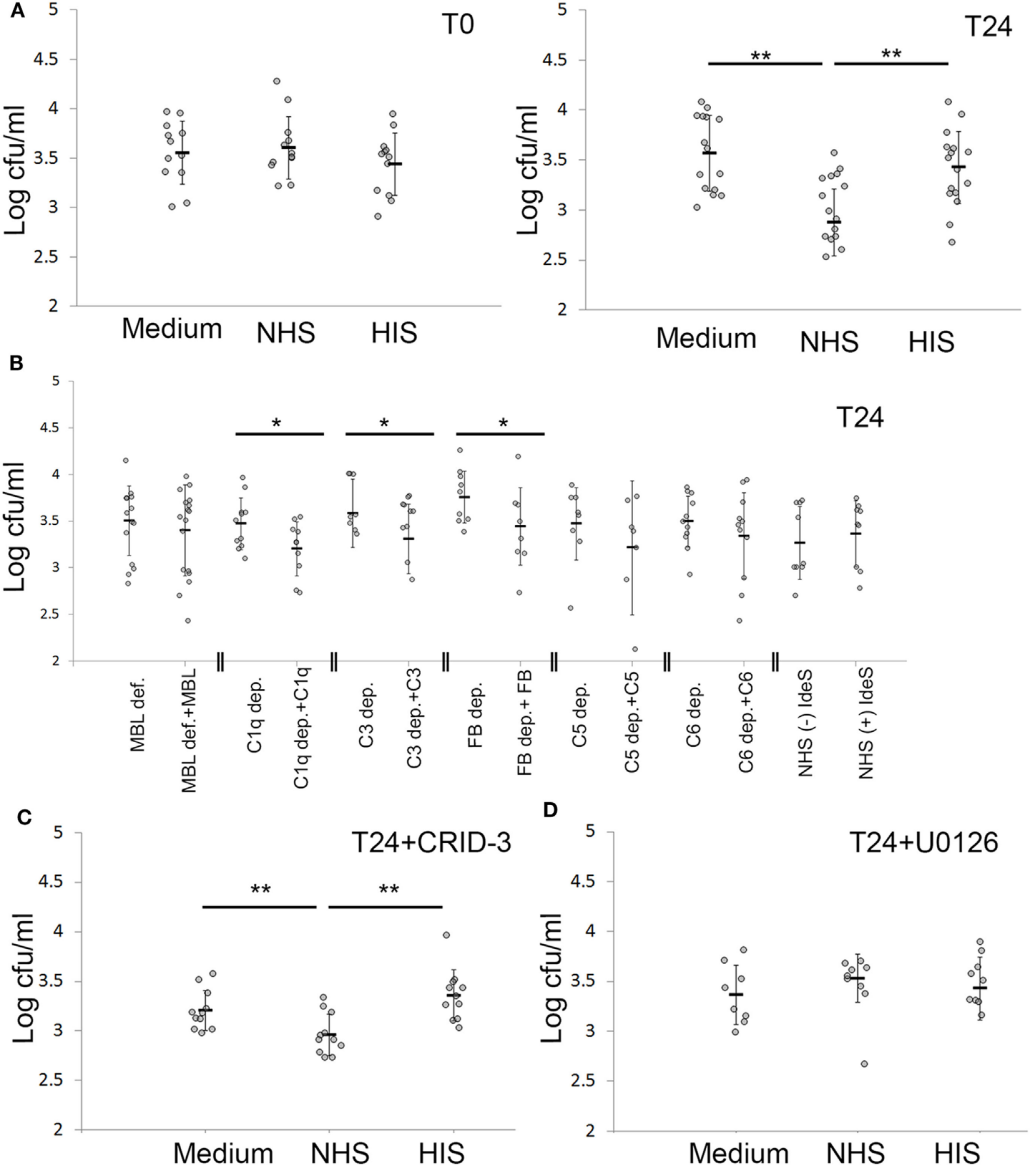
Keratinocytes actively cleared intracellular *Staphylococcus aureus* (SA) after complement activation. **(A)** Keratinocytes infected with SA for 24 h were treated with normal human serum (NHS)/HIS, then lysed and intracellular bacteria plated, either directly after complement activation (T0) or after 24 h (T24). **(B)** Depleted/deficient sera along with the respective reconstitution proteins were used to underline the complement components essential for bacterial clearance. **(C)** The inflammasome inhibitor CRID-3 was used to investigate if the effect seen is related to inflammasome activation, the effect of NHS on viable counts was not changed. **(D)** Treating infected keratinocytes with the ERK inhibitor U0126 abrogated the effect of NHS on bacterial viable counts. Each gray dot represents a keratinocyte monolayer. Black bars represent the average, and error bars represent standard deviation. * *p* < 0.05, ** *p* < 0.01. Each experiment was repeated at least three times, experiments using depleted sera are independent and are only compared with the respective reconstituted serum.

By viable count, we found that NHS treatment of keratinocytes with *persistent intracellular SA* significantly reduced the number of viable intracellular SA compared to HIS or non-treated cells by more than 60% in absolute colony counts (Figure 6B). This reduction of intracellular bacteria was seen 24 h after NHS treatment. No reduction in intracellular bacteria was found directly after NHS treatment (Figure 6A), suggesting that bacterial clearance required a cellular response elicited by NHS treatment.

To pinpoint the role of complement pathways and components important in reducing intracellular bacterial load by NHS treatment, we used depleted and deficient sera of complement components, along with reconstituted sera. We found that reconstitution of C1q, C3 and Factor B depleted sera, but not MBL deficient serum led to a significant decrease in intracellular viable counts (Figure 6B). This demonstrated that complement activation was responsible for the observed reduction in intracellular bacterial load following NHS treatment, and that this effect was mediated by both the classical and alternative pathways but not the MBL pathway. IdeS treatment of NHS did not influence the reduction in intracellular bacteria, indicating that IgG antibodies did not play a major role in the reduction of *persistent intracellular SA* following complement activation (Figure 6B). Reconstitution of C5 and C6 depleted sera also led to reduction of *persistent intracellular SA*; however, this was not statistically significant, demonstrating that TCC was not majorly or solely involved in the reduction of intracellular bacteria, but that activation fragments in particular of C3, played a role in this regard.

### Only Cell Mediated Complement Activation Leads to Reduction in Intracellular Bacterial Load

We then investigated whether only cell mediated complement activation and not complement activation caused by bacterial remnants reduced the intracellular bacterial survival. We have previously demonstrated that EGFR inhibition led to—undefined—cellular responses that cause cell mediated complement activation on the surface of epidermal keratinocytes treated with NHS (28). Thus, EGFR inhibited cells represent a model of keratinocyte mediated complement activation independent of bacteria or extracellular bacterial remnants. Accordingly, we compared intracellular SA viability in cells with *intracellular SA present immediately after infection* where complement activation takes place on bacterial remnants at T0 (Figure 2D), to EGFR-inhibited cells also with *intracellular SA present immediately after infection* at T0, where the later activate complement both on their surface and on the bacterial remnants. We found that 24 h after NHS treatment, EGFR-inhibited cells had significantly less intracellular SA compared to HIS treated cells, unlike non-EGFR-inhibited cells where no significant difference was seen (Figure S3 in Supplementary Material). This demonstrated that only keratinocyte-mediated complement activation, and not complement activation due to extracellular bacterial remnants, caused a reduction in the intracellular bacterial load.

### Complement Mediated Reduction of Persistent Intracellular Bacteria Is a Cellular Response Dependent on Mitogen-Activated Protein Kinase (MAPK) Pathway

To investigate the role of the inflammasome in the killing of *persistent intracellular bacteria*, we inhibited inflammasome activation in infected keratinocytes using the potent inhibitor of nucleotide-binding domain, leucine-rich-containing family, pyrin domain-containing-3 (NLRP3) inflammasome assembly, CRID-3 (39). CRID-3 inhibited interleukin-1 beta release from keratinocytes with *persistent intracellular SA*, demonstrating that CRID-3 inhibited inflammasome assembly in these cells (Figure S4A in Supplementary Material). However, CRID-3 treatment did not influence the number of *persistent intracellular SA* after complement activation (Figure 6C), demonstrating that the complement mediated reduction in *persistent intracellular SA* was not related to inflammasome activation.

To investigate whether the reduction of intracellular bacterial load was related to increased autophagy, we used fluorescence microscopy to detect microtubule-associated protein 1A/1B-light chain 3 (LC3) and lysosomal-associated membrane protein 1 (LAMP-1) and examined the expression of autophagy-related genes Atg5 and beclin-1 using qPCR. We found no significant difference in LC3 or LAMP-1 staining, nor in Atg 5 and beclin-1 expression when comparing cells with intracellular SA treated with NHS or HIS (Figures S4B,C in Supplementary Material). This indicated that the bacterial clearance induced by complement activation was not related to increased autophagy.

However, treatment of keratinocytes harboring *persistent intracellular SA* with U0126, an inhibitor of the MAPKs/extracellular signal-regulated kinases (ERK), abrogated the reduction in the intracellular bacterial load mediated by complement activation (Figure 6D), demonstrating that complement activation reduced intracellular SA by an active cellular process mediated through the MAPK pathway signaling. The effect of U0126 was not due to reduced expression of antimicrobial peptides (AMPs), since we tested human β-defensin 2 (hBD-2), RNase 7, and human β-defensin 3 (hBD-3) and none were induced by complement activation on cells with persistent intracellular bacteria (expression of hBD-2 was not detected) (Figure S4D in Supplementary Material). Furthermore, control experiments using trypan blue staining demonstrated that 10 µM of U0126 or CRID-3 treatment did not affect viability of infected cells (data not shown).

## Discussion

The adept skin pathogen SA has been shown to persist in keratinocytes *in vitro* and *in vivo* (19, 20, 40, 41). The intracellular reservoir is suggested to contribute to dissemination, recurrence and antibiotic resistance (16, 42). While host responses to extracellular SA infections are extensively studied, the host response to intracellular bacterial persistence in non-professional immune cells like keratinocytes is poorly understood. Since persistent intracellular bacterial survival of SA likely affects tissue homeostasis, and the complement system is a pivotal surveillance system of tissue homeostasis, we investigated whether persistent intracellular SA in viable epidermal keratinocytes promoted complement activation. To this end, we set up a model of *persistent intracellular SA* where intracellular bacteria persisted more than 24 h after infection.

We found that primary epidermal keratinocytes with *persistent intracellular SA* activated the complement system. In our model of *persistent intracellular SA*, where extracellular bacteria were killed and removed, we found no co-localization between detectable bacteria/bacterial remnants and deposition of complement, demonstrating that the observed complement activation was not mediated by bacteria/bacterial remnants.

Complement activation takes place on apoptotic and necrotic cells (20, 21). Although keratinocytes have been found to be resilient to SA (18), previous studies have also demonstrated that SA infected keratinocytes can undergo apoptosis after internalization of SA (19). However, we found that complement deposition on keratinocytes with *persistent intracellular SA* in our experimental model was not associated with increased apoptosis or necrosis. Thus, we hypothesize that the persistent presence of viable intracellular bacteria may trigger cellular response(s) or change(s) in cellular homeostasis unrelated to apoptosis or necrosis in a way that leads to complement activation.

Complement activation was performed with sera deficient or depleted of essential complement components to identify the complement pathway responsible for complement activation triggered by the presence of *persistent intracellular SA*. Surprisingly, complement activation was found with both C1q and factor B depleted sera as well as with MBL deficient serum, judged by deposition of C3, C4, and TCC. Experiments with sera where IgG was degraded still demonstrated complement activation, ruling out a major role of IgG even for the C1q-mediated complement activation. This demonstrates that other mechanisms are responsible for the C1q-mediated complement activation like direct binding of C1q and/or natural IgM antibodies to the infected cells (43). Furthermore, a contribution of other recognition molecules like the ficolins and collectins in the lectin pathway could also blur the picture (44). Nonetheless, our data clearly indicate that more than one pathway was involved in the observed complement activation on keratinocytes with *persistent intracellular SA*.

Apart from their role in forming a physical barrier, keratinocytes are active participants in the immune response to skin infections, through production of AMPs, complement components, and chemotactic factors (45, 46). Indeed, we previously found that saliva treatment caused reduction of intracellular SA in infected keratinocytes (31), demonstrating that keratinocytes can respond to external stimuli by actively reducing the number of intracellular bacteria. Consequently, we tested whether the complement activation promoted clearance of *persistent intracellular SA*.

Complement activation on keratinocytes with *persistent intracellular SA* did indeed lead to a reduction in the intracellular bacterial load. Sera depleted of C1q, C3, and factor B, all had significantly decreased the number of intracellular bacteria after reconstitution, while this was not the case for MBL deficient serum. Thus, it seems likely that the complement activation required to decrease the load of intracellular persistent bacteria, was initiated by the classical pathway and amplified by the alternative pathway. No effect on the number of intracellular bacteria was seen directly after complement activation. This was not surprising since the persistent bacteria were intracellular while complement activation took place extracellularly, and also since SA is resistant to direct killing by complement activation (13). Instead, complement activation reduced the number of intracellular bacteria through a cellular response mediated by activated complement components. Complement components have previously been shown to activate keratinocytes either through binding to complement receptors (47) or through sub-lytic TCC insertion (48), and we have previously demonstrated that keratinocytes can reduce the intracellular bacterial load after stimulation (31).

The mechanisms by which keratinocytes reduce the intracellular bacterial load are not well understood, but inflammasome assembly and subsequent caspase-1 dependent cell death or pyroptosis, as well as autophagy, have been suggested as mechanisms to limit survival of intracellular pathogens (49). However, inhibition of inflammasome assembly did not reduce the number of *persistent intracellular SA*, and complement activation did not induce autophagy. We previously found that saliva (as a model of wound licking) reduced the number of intracellular bacteria in keratinocytes, paralleled with expression of AMPs, especially human β-defensin-3 (31). Yet, complement activation did not induce expression of human β-defensin-3 or other AMPs. The MAPK pathway is important for regulating immune functions in keratinocytes (50, 51). Inhibition of MAPK dependent ERK activation abrogated the complement mediated reduction of *persistent intracellular SA*, thus demonstrating that the MAPK pathway is involved in complement mediated cellular responses which reduce the load of persistent intracellular SA.

Although cells with intracellular bacteria have been demonstrated to undergo autophagy, apoptosis and pyroptosis (52), keratinocytes have been found to be resilient to SA (53). Nevertheless, our data demonstrate that keratinocytes seemed to adjust to the persistent intracellular SA with subtle and not yet identified changes that in turn promote complement activation, not unlike what is seen after EGFR inhibition (28). To understand the role of the complement system in the dynamic interplay between bacteria and keratinocytes in chronic and therapy resistant infections, the cellular changes or adaptions to the persistent intracellular bacteria that result in complement activation should be further delineated.

The complement system is known to have important roles in the skin. Locally synthesized complement components in the skin play a role in inflammatory skin diseases (54, 55), and complement deficiencies are associated with pyogenic skin infections (4). Blocking of certain complement proteins alters the skin microbiome (56). Since plasma exudation commonly follows epidermal inflammation, we hypothesize that complement activation induced by keratinocytes with persistent intracellular bacteria could maintain an inflammatory response after clearing the initial insult while at the same time limiting the number of intracellular bacteria. This could be relevant in AD patients commonly infected with SA during AD flares. Although the contribution of intracellular SA in AD has not been examined, and is beyond the scope of this work, our *in vivo* data suggest that intracellular SA in the epidermis could lead to activation of complement, thereby contributing to the inflammation found in SA-colonized AD skin. In this regard, it is worth noting that anti-staphylococcal therapies may play a role in severe, superinfected AD (57). Although there is no clear evidence for a clinical benefit of antibiotic therapies in non-infected AD, short courses of systemic antibiotics in more extensive forms of superinfected AD might be needed (9, 57, 58). If complement activation induced by *persistent intracellular SA* plays a role in the inflammation seen in AD flares, it would probably be beneficial if the chosen antibiotic treatment targets not only extracellular bacteria but also the *persistent intracellular SA*.

The complement system is seen as an important surveillance system for maintaining tissue homeostasis. Our study demonstrates that keratinocytes with persistent intracellular SA activate the complement system resulting in reduction of the intracellular bacterial load. This ties together the known functions of the complement system in host defense and tissue homeostasis and demonstrates a novel role of the complement system in combating bacteria like SA, otherwise resistant to complement mediated killing, when present intracellularly in non-phagocytic cells.

## Ethics Statement

This study was carried out in accordance with the recommendations of the Regional Ethics Examination Board of Lund, Sweden. All subjects gave written informed consent in accordance with the Declaration of Helsinki. The protocol was approved by the Regional Ethics Examination Board of Lund, Sweden (Permit Numbers: 144/2010, 317/2010, 82/2012).

## Author Contributions

AA-H contributed in experimental design, data acquisition, data analysis and interpretation, writing, and revision of the manuscript. ME, AS, and PG contributed to data acquisition, writing and revision of the manuscript. OS contributed to the study conception, experimental design, data interpretation, writing, and revision of the manuscript.

## Conflict of Interest Statement

The authors declare that the research was conducted in the absence of any commercial or financial relationships that could be construed as a potential conflict of interest.
