# Supplementary material for: Persistent Intracellular Staphylococcus aureus in Keratinocytes Lead to Activation of the Complement System with Subsequent Reduction in the Intracellular Bacterial Load
Source: Front Immunol. 2018 Mar 1;9:396. doi: 10.3389/fimmu.2018.00396 (PMC5837974; doi:10.3389/fimmu.2018.00396)
Supplement: Supplementary file 1 [file image_1.PDF]

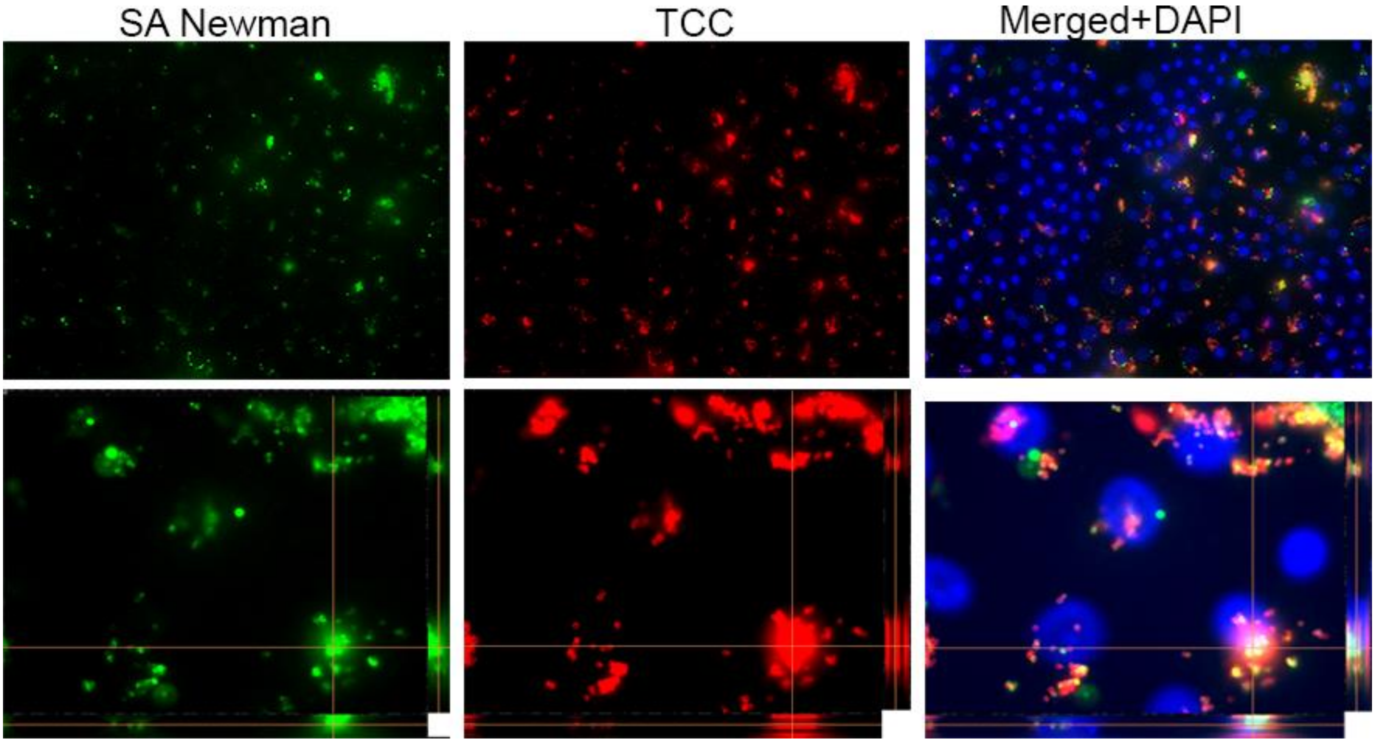

Supplementary figure 1. Immunofluorescence microscopy of keratinocytes infected with SA Newman (green) - defective in host cells invasion - and TCC (red). Orthogonal views of Z-stacks in the lower panel show high colocalization of TCC and SA, indicating complement activation on extracellular SA Newman.

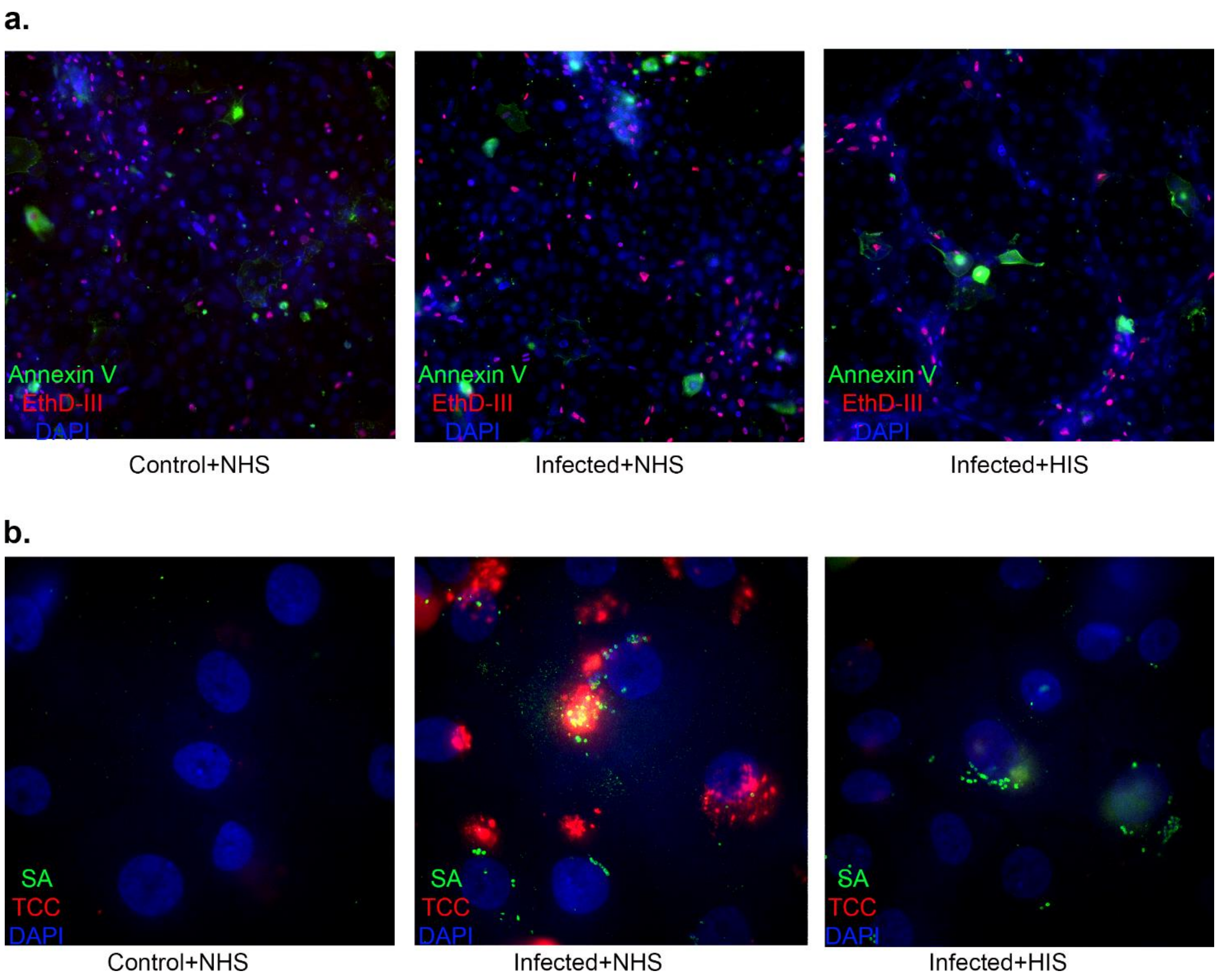

Supplementary figure 2. Immunofluorescence microscopy of keratinocytes infected with persistent intracellular SA after 7 days of initial infection. (a) Representative images of an apoptosis/annexin-V (green) and necrosis/ Eth-III (red) assay that show generalized apoptosis and necrosis in infected and non-infected monolayers. (b) Infected keratinocytes with persistent intracellular SA (green) after 7 days of initial infection, still activate complement as seen by deposition of TCC (red) on the surface of infected keratinocytes when incubated with NHS.

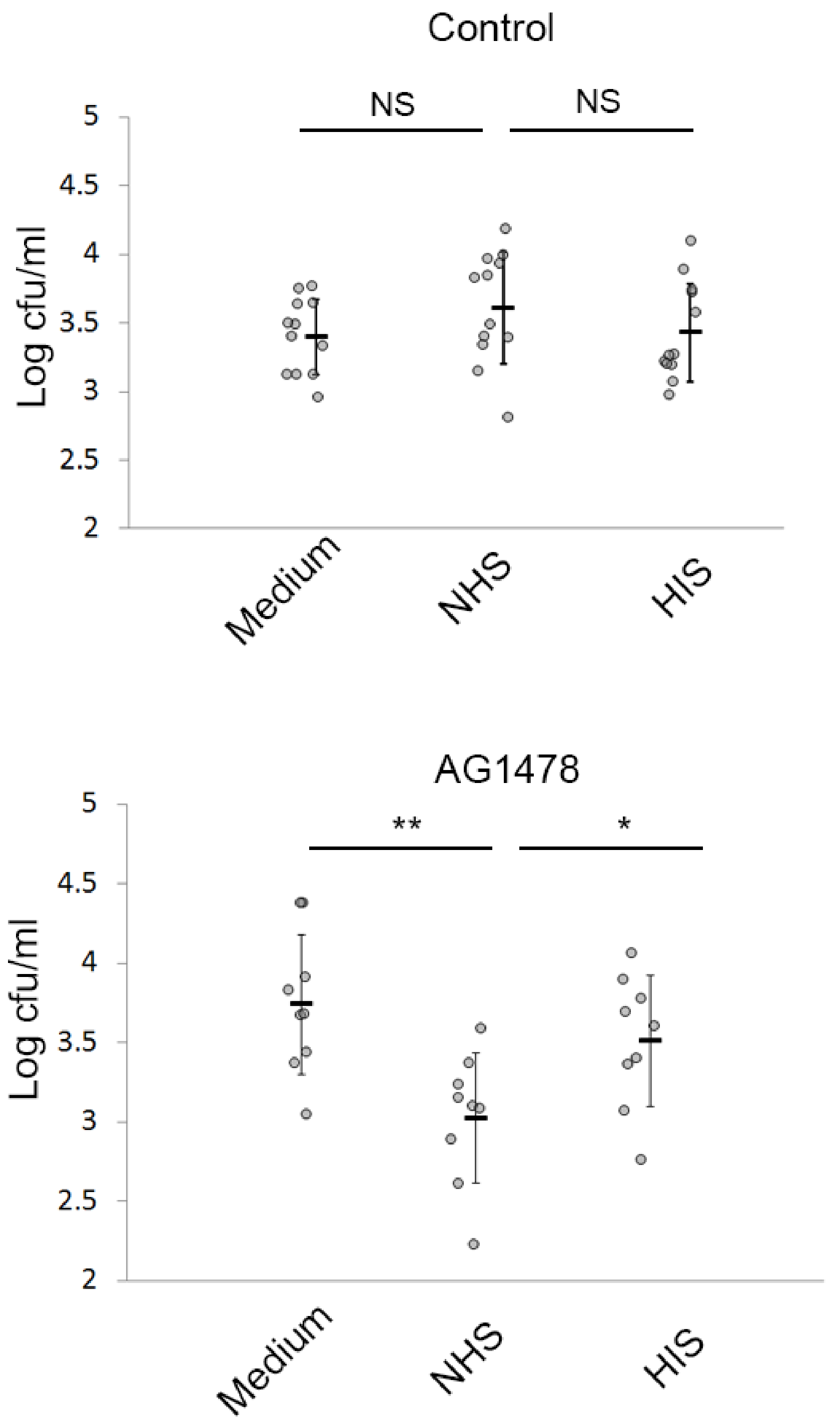

Supplementary figure 3. Viable counts of intracellular SA after 24 hours from complement activation. EGFR-inhibited keratinocytes (using the tyrosine kinase inhibitor AG1478) have been shown to activate complement on their surface, unlike untreated keratinocytes (control). A significant decrease in viable counts following incubation with NHS was found in AG1478 treated cells, unlike controls.

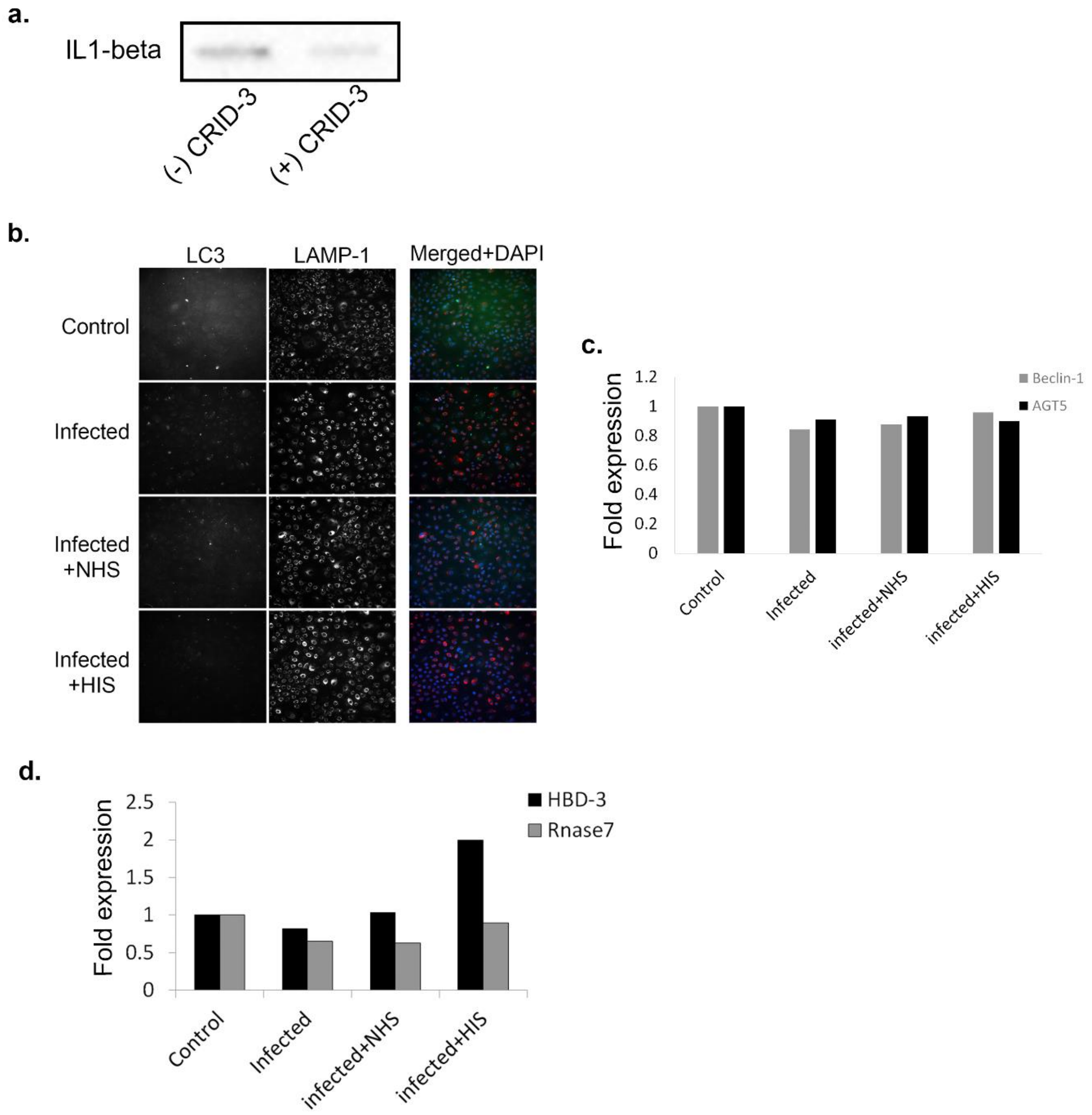

Supplementary figure 4. Investigating the pathway responsible for clearance of persistent intracellular SA following complement activation(a) Western blot of IL-1 beta in medium of cells treated with CRID-3, an inhibitor of inflammasome assembly, show a decrease in IL1-beta secretion in the medium. (b) Representative mmunofluorescence microscopy images of LC3 (green) and LAMP-1 (red) show no increase in LC3, present on autophagosomes, or LAMP-1, present on lysosomal membranes, following NHS treatment, indicating that autophagy is probably not responsible for clearance of persistent intracellular SA following complement activation. (c) Autophagy role was further investigated using real time PCR, we found no significant change following NHS treatment in expression of ATG5 or Beclin-1, both proteins important in autophagy. (c) Expression of ERK related defensins HBD-3 and RNase7, show no significant increase in expression following NHS treatment, indicating that defensins are not responsible for clearance of persistent intracellular SA following complement activation, expression of HBD-2 was not detected in control or infected cells.
